# Using routinely collected primary care records to identify and investigate severe asthma: a scoping review

**DOI:** 10.1038/s41533-020-00213-9

**Published:** 2021-01-26

**Authors:** Jonathan Stewart, Frank Kee, Nigel Hart

**Affiliations:** 1 Centre for Public Health, Queen’s University Belfast, Institute of Clinical Science, Block A, Royal Victoria Hospital, Belfast, BT12 6BA UK; 2 Centre for Medical Education, Queen’s University Belfast, Whitla Medical Building, 97 Lisburn Road, Belfast, BT9 7BL UK

**Keywords:** Asthma, Diagnosis

## Abstract

Shielding during the coronavirus pandemic has highlighted the potential of routinely collected primary care records to identify patients with ‘high-risk’ conditions, including severe asthma. We aimed to determine how previous studies have used primary care records to identify and investigate severe asthma and whether linkage to other data sources is required to fully investigate this ‘high-risk’ disease variant. A scoping review was conducted based on the Arksey and O’Malley framework. Twelve studies met all criteria for inclusion. We identified variation in how studies defined the background asthma cohort, asthma severity, control and clinical outcomes. Certain asthma outcomes could only be investigated through linkage to secondary care records. The ability of primary care records to represent the entire known asthma population is unique. However, a number of challenges need to be overcome if their full potential to accurately identify and investigate severe asthma is to be realised.

## Introduction

The majority of asthma care in the United Kingdom (UK) is delivered in primary care. Severe asthma represents a subset of patients whose disease does not respond to treatment in this setting and remains uncontrolled despite confirmed adherence with maximal optimised therapy and treatment of contributory factors or that worsens when high-dose treatment is reduced^1^. This is distinct from difficult-to-treat asthma (DTA), where poor control is due to modifiable factors, such as incorrect inhaler technique, poor adherence, smoking, comorbidities or an incorrect diagnosis.

Patients with severe asthma have significantly better outcomes when identified and referred for specialist assessment^2^. There is significant variation in asthma specialist care across the UK, with unacceptable variation in prevalence, frequency of exacerbations, provision of services and health outcomes across geography, age, ethnicity and socio-economic groups^3,4^. New safe and effective management options are available for severe asthma, and it is vital that we better understand what contributes to this variation and put in place measures to reduce its effect.

Severe asthma was named as one of the ‘high-risk’ conditions during the coronavirus outbreak^5,6^. Searches of routinely collected primary care records were conducted in an attempt to rapidly identify these patients and advise them to isolate themselves to prevent harm from contracting the virus. This has placed a spotlight on the challenges of accurately identifying patients with severe asthma from routinely collected data, including gaining consensus on what criteria should be used to define this subgroup^7^. This process has also highlighted exciting potential opportunities such as improving our understanding of this high-risk disease, gaining accurate estimates of prevalence and disease burden and identifying potential candidates for novel therapies.

This aim of this review was to identify how previous studies have used primary care data to identify and investigate severe asthma. Given that asthma patients have healthcare records held in various other databases throughout the Health and Social Care system, we also aimed to determine the benefits and limitations of linking primary care data to other healthcare and administrative data.

## Results

**Fig. 1:**
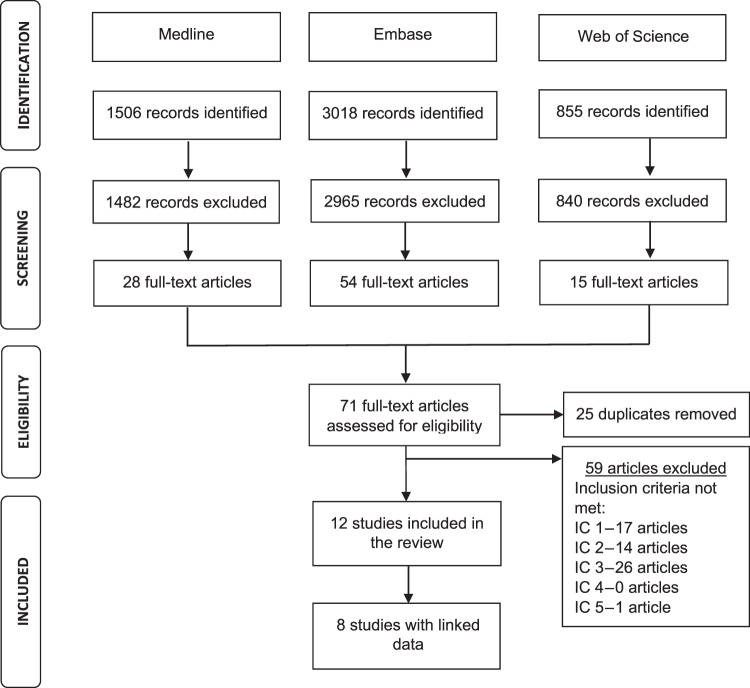
Preferred Reporting Items for Systematic Reviews and Meta-Analyses (PRISMA) flowchart for study selection. IC inclusion criteria, IC 1 routinely collected primary care records, IC 2 primary care asthma population, IC 3 varying severities of asthma identified. IC 4 full peer-reviewed article available. IC 5 English.

One thousand five hundred and six records were identified from Ovid Medline, 3018 records from Embase and 855 from Web of Science (Fig. 1). Following screening, 28 full-text articles were identified from OVID Medline, 54 from Embase and 15 from Web of Science. After removal of duplicates, 71 full-text articles were included in the full review stage. Twelve studies met all the inclusion criteria. Of these, 8 studies linked General Practice (GP) data to other healthcare and/or administrative records.

### Data sets

Of the 12 studies included in the review, 9 obtained primary care data from the UK^8–15^, 1 from Sweden^16^, 1 from Denmark^17^ and 1 from the United States of America (USA)^18^ (Table 1). The UK studies obtained primary care data from 5 sources of varying size and coverage of the UK population. The Swedish study obtained primary care data from a cluster of Swedish primary medical centres^16^. The Danish and American studies primarily obtained primary care data from Health Insurance registers.

**Table 1:** Primary care, secondary care and administrative data sets used by the included studies.

	Gayle	Price 2015	Turner	Walsh	Yang	Nissen	Bloom	Price 2016	Hull	Larsson	Moth	Shields
*Primary care data sets*												
General Practice data sets	X	X	X	X	X	X	X	X	X	X		
CPRD	X	X	X			X	X	X				
OPCRD		X	X					X				
Nottingham General Practices				X								
North Glasgow EMIS General Practices					X							
North East London EMIS General Practices									X			
Sweden Primary Care Centres										X		
Health Insurance Registers											X	X
Danish Health Insurance											X	
Massachusetts Medicaid												X
*Secondary care and administrative data sets*												
Hospital Databases					X	X	X	X	X			
NHS Health Board Records					X							
UK HES						X	X	X				
UK SUS									X			
Community Prescribing Register					X							
National Registers										X	X	
Swedish										X		
Danish											X	
Health Insurance Databases												X
Massachusetts Medicaid												X

*CPRD* Clinical Practice Research Datalink, *HES* Health Episode Statistics, *SUS* Secondary Uses Services, *NHS* National Health Service, *ONS* Office of National Statistics, *OPCRD* Optimum Patient Care Research Datalink, *UK* United Kingdom.

Eight studies linked primary care data to other healthcare or administrative data^8–11,16–18^ (Table 1). UK secondary care data was obtained from sources including hospital databases^8–11^. The remaining articles obtained secondary care data from national registers and health insurance registers^17,18^.

### Asthma cohorts

Asthma was defined by either an asthma ‘read code’^8–15^ or prescription records for asthma or obstructive airways disease medications^16–18^ (Table 2). Included articles had variable inclusion and exclusion criteria. Inclusion criteria included an available eosinophil count^8,10,13,14^, Body Mass Index (BMI)^8^, smoking status^8,13^ or data linkage to specified secondary care and administrative databases^8,9^. Exclusion criteria included another chronic respiratory condition^10,13,14^, conditions associated with oral corticosteroid (OCS) use^16^ or a very high eosinophil count (>5000/µL, to avoid extreme outliers)^10,14^. Seven studies included adults and children^9–13,15,19^, 2 studies included only children^17,18^ and 2 studies included only an adult population^8,16^. Final asthma cohorts varied in size from <10,000 patients^14,15,18,19^, to 10,000–100,000 patients^11,17^ to >100,000 patients^8–10,12,13^.

**Table 2:** Definitions, inclusion and exclusion criteria and size of asthma cohorts.

	Gayle	Price 2015	Turner	Walsh	Yang	Nissen	Bloom	Price 2016	Hull	Larsson	Moth	Shields
*Asthma cohort definition*												
Asthma read code	X	X	X	X	X	X	X	X	X			
Prescription for asthma											X	X
Prescription for obstructive pulmonary disease										X		
Specialist confirmed asthma										X		
Asthma hospitalisation												X
Asthma ambulatory visits												X
*Inclusion criteria*												
Specified period of follow-up	X	X	X			X		X				
Age (years)	5–80		5–12	≥4		≥18		12–80	5–75	≥18	6–14	2–18
Asthma prescription in previous year					X				X			
Eosinophil count		X	X			X		X				
BMI recorded						X						
Smoking status recorded		X				X						
Linked HES/ONS data						X	X					
Specified health insurance enrolment												X
*Exclusion criteria*												
Chronic respiratory disease (other than asthma)		X	X					X				
RA and/or PMR										X		
Moved country during observation period											X	
Very high eosinophil count (>5000/µL)			X					X				
Inclusion in clinical trial	X											
*Asthma cohort size*												
Asthma cohort available in the database	796K	406K	634K	UA	4K	766K	933K	406K	36K	49K	46K	11K
Asthma cohort included in the study (met entry criteria)	647K	130K	4K	3K	2K	194K	424K	131K	36K	17K	37K	6K
Proportion of asthma cohort included in the study (%)	81.3	32.0	0.6	UTC	50	25.3	45.5	45.5	100	33.8	86.7	58.3

*COPD* chronic obstructive pulmonary disease, *HES* Hospital Episode Statistics, *ONS* Office of National Statistics, *PMR* polymyalgia rheumatica, *RA* rheumatoid arthritis, *UA* unavailable from article, *UTC* unable to calculate.

### Asthma severity and control

Varying severities of asthma were defined by British Thoracic Society (BTS) treatment steps^8–13,15^ or Global Initiative for Asthma (GINA) treatment steps^14,16^ (Table 3). One article defined severity by exacerbation frequency^18^ and one article defined severity by frequency of anti-asthma medication prescriptions^17^. For studies that classified severity using treatment steps, the proportion of patients reported with steps consistent with severe asthma ranged from 2 to 22% for Step 4 and from 0.2 to 1% for Step 5^8,10–13,15,19^ (Supplementary Tables 1a, b).

**Table 3:** Definitions of asthma severity, control and clinical outcomes.

	Gayle	Price 2015	Turner	Walsh	Yang	Nissen	Bloom	Price 2016	Hull	Larsson	Moth	Shields
*Asthma severity*												
BTS treatment steps	X	X		X	X	X	X	X	X			
BTS 2016					X	X	X		X			
BTS 2014	X	X										
BTS 2011								X				
BTS 1995				X								
GINA treatment steps			X							X		
Exacerbation frequency												X
Anti-asthma prescription frequency											X	
*Asthma control*												
Exacerbation frequency				X	X	X	X	X				
SABA use				X	X	X		X	X	X		X
Combined measure		X	X							X		
*Clinical outcomes*												
Exacerbations (ATS/ERS definition)		X	X					X		X		
ED visits		X	X		X	X	X	X		X		X
Hospitalisations		X	X		X	X	X	X	X	X		X
OCS prescriptions		X	X	X	X	X	X	X		X		
GP visits							X			X		X
Asthma deaths						X	X					

*ATS/ERS* American Thoracic Society/European Respiratory Society, *BTS* British Thoracic Society, *ED* emergency department, *GINA* Global Initiative for Asthma, *GP* General Practice, *OCS* oral corticosteroid, *SABA* short-acting beta agonist.

Asthma control was defined by exacerbation frequency and short-acting beta agonist (SABA) reliever inhaler overuse (Table 3). The proportion of patients with excessive SABA use ranged from 9.1 to 13.6% when defined by inhaler prescriptions per year (≥10 or ≥13 prescriptions)^8,11,15,19^ and from 22% to 23.5% when by defined by SABA daily dosage (≥300 or ≥400 µg)^10,14^ (Supplementary Tables 2a, b). Three articles used combined measures of asthma control^13,14,16^ (Table 3). Two articles that used comparable measures of ‘overall asthma control’ estimated the proportion of patients with uncontrolled asthma at 26.7 and 59.3%^13,14^ (Supplementary Tables 2a, b).

### Clinical outcomes

Asthma exacerbations were the most commonly measured asthma outcome (Table 3). Six articles used the American Thoracic Society/European Respiratory Society (ATS/ERS) definition for an asthma exacerbation (asthma-related emergency department (ED) visit, hospitalisations or OCS prescription)^8–10,13,14,16^. Three articles that used this definition of asthma exacerbations found that the proportion of patients with >2 exacerbations per year ranged from 5 to 7%^10,13,14^ (Supplementary Tables 3a, b). The remaining articles used OCS prescriptions^15,19^, hospitalisations^11^ or both^18^ (Table 3). Two articles included GP visits for asthma^18,20^ and two included death due to asthma^8,20^ in their definitions of an asthma exacerbation.

### Other themes

We identified four other recurring themes: how the studies characterised their cohorts, what comorbidities they took note of and analysed and if they reviewed healthcare resource utilisation (HCRU) or the quality of care provided (Table 4). We grouped parameters used to characterise the asthma population into socio-demographic factors, investigations and management. The most common socio-demographic parameters were age, sex, BMI and smoking status. Other parameters included ethnicity^11,18^ and socio-economic status^8,9,12,17,19^. Clinical investigations reported included eosinophil counts^8,10,13,14^ and respiratory function using peak flow^10,12–14^ and spirometry^12,13,16^. A number of studies described the asthma treatment received by their cohort including SABA and inhaled corticosteroid (ICS) prescriptions^8,10,13,15–19^. Three articles expanded this to other asthma treatments^16–18^, and one article reviewed non-asthma treatment^16^.

**Table 4:** Recurring themes analysed in the included articles using available data.

	Gayle	Price 2015	Turner	Walsh	Yang	Nissen	Bloom	Price 2016	Hull	Larsson	Moth	Shields
*Demographic characteristics*												
Sex	X	X	X	X	X	X	X	X	X	X	X	X
Age	X	X	X	X	X	X	X	X	X	X	X	X
BMI/obesity		X	X			X	X	X		X		
Smoking status					X	X	X	X	X			
Ethnicity									X			X
Geographic location	X										X	
Socioeconomic status				X		X	X				X	X
Deprivation quintile						X	X					
Townsend Deprivation Score				X								
Parental income/education											X	
Disability												X
*Investigations*												
Eosinophil count			X			X		X		X		
Peak flow	X	X	X					X				
Spirometry	X	X								X		
*Clinical management*												
Asthma treatment step		X			X	X		X	X			
SABA		X		X	X	X		X		X	X	X
ICS		X		X	X			X		X	X	X
LABA										X	X	X
ICS/LABA combination										X	X	X
LTRA										X	X	
Anti-IgE monoclonal antibody										X		
LAMA										X		X
OCS							X	X		X		X
Antibiotics for LRTIs		X	X							X		
Antihistamines										X		
Nasal steroids										X		
Anti-dyslipidaemia										X		
Antihypertensive										X		
Beta blocker								X		X		
Antidepressant										X		
Hypnotics										X		
Antianxiety										X		
Bisphosphonates										X		
Calcium/vitamin D										X		
Paracetamol								X				
NSAID								X				
*Healthcare resource utilisation*												
GP/physician visit							X		X	X		X
Primary care specialist											X	
ED							X	X		X	X	X
Outpatient										X		X
Hospital admission							X	X	X	X		X
*Quality of care measures*												
Access to specialist										X		X
Suboptimal prescribing												
Overprescribing SABA									X			X
Under prescribing ICS									X			
Anti-inflammatory use												X
Adherence to guidelines												
Asthma review in the past year		X			X				X			
Inhaler technique checked									X			
Lung function monitoring		X									X	
Follow-up post-treatment change		X									X	
Follow-up post-exacerbation												X
*Comorbidities*												
Number of comorbidities					X							
Charleston Comorbidity Index		X						X				
Atopy						X	X			X		
Hay fever	X		X									
Eczema	X	X	X		X			X				
Rhinitis	X				X		X	X				
Sinusitis												
Nasal polyps										X		
Anaphylaxis history								X				
COPD	X					X	X		X	X		
Acute bronchitis												
Chronic bronchitis										X		
GORD					X	X	X	X				
Acute URTI										X		
Acute LRTI			X				X			X		
Pneumonia										X		
Influenza										X		
Anxiety					X	X	X	X				
Depression					X	X	X	X				
Diabetes		X			X							
Type 1 diabetes										X		
Type 2 diabetes										X		
Diabetes								X				
Osteoporosis					X					X		
Ischaemic heart disease					X			X		X		
Heart failure								X		X		
Cerebrovascular disease										X		
Hypertension					X					X		
Psoriasis								X				
Inflammatory bowel disease										X		
Malignant neoplasm										X		

*ATS* American Thoracic Society, *BMI* Body Mass Index, *ED* emergency department, *ERS* European Respiratory Society, *FEV1* forced expiratory volume in first second, *FVC* forced vital capacity, *GP* General Practice, *ICS* inhaled corticosteroid, *IgE* immunoglobulin E, *ICU* Intensive Care Unit, *LABA* long-acting beta agonist, *LAMA* long-acting muscarinic antagonist, *LRTI* lower respiratory tract infection, *LTRA* leukotriene receptor antagonist, *NSAID* non-steroidal anti-inflammatory drug, *OCS* oral corticosteroid, *SABA* short-acting beta agonist, *URTI* upper respiratory tract infection.

Eight articles characterised their cohorts by specific comorbidities^8–10^ (Table 5). Two articles included summary measures of comorbidities^13,15^. HCRU including GP visits, ED visits and hospital admissions was analysed by six articles^9–11,16–18^. Seven articles included measures of quality of care, including access to an asthma specialist^18^, suboptimal prescribing^8,11,18^ or adherence to guidelines^11,17,18^.

**Table 5:** Comparison of the quality of study reporting using the RECORD extension to the STROBE statement checklist.

			Gayle	Price 2015	Turner	Walsh	Yang	Nissen	Bloom	Price 2016	Hull	Larsson	Moth	Shields
*Title and abstract*														
Introduction	1	(a) Study design		X	X	X		X	X	X		X	X	X
		(b) Abstract	X	X	X	X	X		X	X	X	X	X	X
		R 1.1 Type of data & database names	X	X	X	X	X	X	X	X	X	X	X	X
		R 1.2 Geographic region & timeframe	X	X		X	X	X	X	X	X	X		X
		R 1.3 Database linkage	1	1	1	1		X	X		X	X	X	X
Background/rational	2		X	X	X	X	X	X	X	X	X	X	X	X
Objectives	3		X	X	X	X	X	X		X	X	X	X	X
*Methods*														
Study design	4		X	X	X	X	X	X	X	X		X	X	X
Setting	5		X	X	X	X	X	X	X	X	X	X	X	X
Participants	6	(a) Participants	X	X	X	X	X	X	X	X	X	X	X	X
		(b) Matched studies	3	3	3	3	3	3	3	3	3	3	3	3
		R 6.1 Study population selection	S	X	X		X	X	X	X	X	X	X	X
		R 6.2 Validation of codes/algorithms	X					X	X			X	X	
		R 6.3 Data linkage process flow diagram	1	1	1	1								
Variables	7		X	X	X		X	X	X	X	X	X	X	X
		R 7.1 Complete code/algorithm list	S		S		S	S	X			X	X	
Data sources/measurement	8		X	X	X	X	X	X	X	X	X	X	X	X
Bias	9												X	
Study size	10		X	X	X		X	X	X	X	X	X	X	X
Quantitative variables	11		X	X	X		X	X	X	X	X	X	X	X
Statistical methods	12	(a) Methods	X	X	X	X	X	X	X	X	X	X	X	X
		(b) Subgroups/interactions	X	X	X	X	X	X	X	X	X	X	X	X
		(c) Missing data	X		X				X	7				
		(d) Loss to follow-up or matching	X	2	2			X						
		(e) Sensitivity analyses	X					X	X			X		
		R 12.1 Access to database population	X	X	X	X	X	X	X	X		X	X	X
		R 12.2 Data cleaning methods									X			
		R 12.3 Data linkage level, methods and quality evaluation	1	1	1	1					X			
*Results*														
Participants	13	(a) Numbers	S	X	X		X	X	X	X	X	X	X	
		(b) Non-participation	S	X	X		X	X	X	X		X	X	
		(c) Flow diagram	S	X	X			X	X	X		X		
		R 13.1 Study population selection	X	X	X			X	X			X		
Descriptive data	14	(a) Characteristics	X	X	X		X	X	X	X	X	X	X	X
		(b) Missing data			X				X	6	X			X
		(c) Follow-up (average or total)		2	2			X	X	2				
Outcome data	15		X	X	X	X	X	X	X	X	X	X	X	X
Main results	16	(a) Unadjusted & adjusted	X	X	X	X	X	X		X		X	X	X
		(b) Category boundaries	4	X	X	4	X	X		X	X	4	X	X
		(c) Relative risk to absolute risk	5			5		X	X					X
Other analyses	17	Subgroups, interactions or sensitivity	X	X	X	X	X	X	X	X	X	X	X	X
*Discussion*														
Key results	18		X	X	X	X	X	X	X	X	X	X	X	X
Limitations	19	Study limitations	X	X	X	X	X	X	X	X	X	X	X	X
		R 19.1 Data limitations	X	X	X		X	X	X	X	X	X	X	X
Interpretation														
Interpretation	20		X	X	X	X	X	X	X	X	X	X	X	X
Generalisability														
Generalisability	21		X	X	X	X		X	X	X	X	X	X	X
*Other information*														
Funding	22		X	X	X	X	X	X		X	X	X	X	X
		R 22.1 Study protocol, raw data or programming code	X		X		X	X	X		X			
*Reporting summary*														
Study reporting		Checklist points covered	34	31	35	21	30	40	37	31	29	35	33	31
		Valid points available	41	41	41	41	46	46	46	43	46	45	46	46
		Proportion of valid points (%)	82.9	75.6	85.4	51.2	65.2	87.0	80.4	72.1	63.0	77.8	71.7	67.4

*R*—RECORD criteria; S—information available from supplementary materials of article; 1—Not applicable: no data linkage conducted; 2—Not applicable: inclusion criteria specified complete data for follow-up period available, therefore no loss to follow-up for final cohort; 3—Not applicable: study did not involve matching; 4—Not applicable: no categories in output data; 5—Not applicable: no risk analysis included; 6—Not applicable: inclusion criteria specified complete baseline and outcome data.

### Reporting of studies

We reviewed the quality of reporting of studies against the REporting of studies Conducted using Observational Routinely-collected health Data (RECORD) extension to the Strengthening the Reporting of Observational Studies in Epidemiology (STROBE) Statement^21–23^. The most poorly reported areas of STROBE Statement areas (less than five articles) were explanations of efforts taken to address a source of bias^17^, how missing data were addressed^9,12,14^ and the number of participants with missing data for variable of interest^9,11,18^.

For the RECORD extension areas, four articles provided the required detail on how codes or algorithms used to identify the study population were validated^8,9,16,17^ and none of the included articles provided a description of the data cleaning methods. For the articles that linked to other data sets, none of the included articles provided a data linkage flow diagram or provided the required detail on data linkage level, methods and quality evaluation. When the quality of study reporting was compared numerically using the RECORD criteria, 5 articles covered 80% of more of the required checklist areas^9,12,14,16,24^.

## Discussion

The majority of asthma care in the UK is carried out in primary care, yet a significant proportion of previous research into this condition has taken place outside this setting. A previous review of the use of electronic health record-derived data to define asthma and assess asthma outcomes identified a number of limitations of the included studies, the majority of which used data extracted from secondary care^25^. This aim of this review was to identify how previous studies have used primary care data to identify and investigate severe asthma. We have summarised some of the challenges identified from reviewed articles for each step in the identification of patients with severe asthma, the potential opportunities if we can accurately identify these patients and propose how identified challenges might be overcome to realise these opportunities.

Before attempting to identify patents with severe asthma, the first challenge is to agree on how to identify the background asthma population from primary care records, within which patients with severe disease can be identified. The majority of articles included in the review used asthma ‘read codes’ to identify patients with asthma from primary care records. Remuneration in UK primary care via the Quality and Outcomes Framework (QOF) requires the use of these specific read codes^26^. GPs are required to hold, and to keep up to date, an accurate asthma patient population. Nissen et al. reported that the UK has benefitted from higher-quality coding due to QOF, and asthma can be accurately identified from UK primary care records using specific read codes, with a high positive predictive value for asthma (86%)^24^. By contrast, using medication records from primary care to identify patients with asthma has limitations, most notably the potential to miscategorise patients with conditions who use similar medications, in particular chronic obstructive pulmonary disease (COPD)^9,20^.

A number of the included studies had pre-specified inclusion and exclusion criteria to identify their asthma cohorts. This has advantages if researchers want to investigate specific known or novel associations, such as with eosinophilia^8,10^. Excluding patients with COPD and other chronic respiratory disease can reduce miscategorisation^9,16^. However, these inclusion and exclusion criteria, required to address the specific objectives of each study, will reduce the generalisability of study findings to the wider asthma population in primary care and limit the ability to compare findings between studies^16^.

Once the background asthma population is identified, the next challenge is to agree on the criteria to diagnose ‘severe’ asthma. The majority of articles included in this review categorised the severity of their primary care asthma populations using prescribing records into the BTS^8–13^ or GINA^14,16^ treatment steps. The GINA 2017 guideline defines treatment Steps 4 and 5 as severe asthma^27^. Despite differences in the earlier treatment steps between guidelines, Steps 4 and 5 are essentially the same across BTS and GINA guideline versions (Supplementary Table 3)^28,29^. If we compare BTS and GINA guidelines to the definition of severe asthma used to identify patients with ‘high risk’ severe asthma during the coronavirus pandemic^5^, BTS and GINA Step 5 treatment would be required to meet the criteria or lower steps with admission to hospital or the Intensive Care Unit (Supplementary Table 3). Prescribing records for primary care records are amendable to categorisation by these treatment steps. However, this is not without challenges. Asthma guidelines change regularly, making comparison between studies difficult. Since the included studies were published, the BTS and GINA guidelines have both been updated, making comparison with future studies even more challenging (Supplementary Table 3)^28,29^.

Under the GINA classification, for an accurate diagnosis of severe asthma the disease must remain uncontrolled despite adherence to maximal treatment and management of modifiable contributory factors^1^. Therefore, the next challenge is to determine whether a patients’ asthma is poorly controlled. In the studies included in this review, asthma control was generally defined by SABA reliever inhaler use and/or frequency of exacerbations. SABA overuse is a well-recognised surrogate measure of poor asthma control. The National Review of Asthma Deaths highlighted excessive SABA prescriptions as a predictor of poor outcomes, including asthma-related death^30^. While data on high levels of SABA prescribing is readily available from primary care prescription records in the UK, it is unclear whether the patient is actually using all prescribed treatment and whether inhalers are being used with the correct technique.

The ATS/ERS Task Force defines an asthma exacerbation as an OCS prescription, ED visit or hospitalisation^31^. As for SABA overuse, short courses of OCSs can be readily extracted from primary care data. However, their accuracy as an indirect measure of asthma exacerbations has limitations. A previous study found that one-third of patients with an unscheduled visit for asthma did not receive an OCS course^32^. Furthermore, short courses of OCS are prescribed for a variety of conditions other than asthma, and it can be difficult to determine which condition they were prescribed for, potentially overestimating asthma exacerbation frequency.

Accurate assessment of ED visits and hospitalisations requires linkage of primary care records to other secondary care data sets. Price et al. acknowledged that hospital admissions tend to be underreported in the primary care databases Optimum Patient Care Research Database and Clinical Practice Research Datalink, and for this reason they linked to the Hospital Episode Statistics secondary care database^10^. This raises the question of how accurate measurement of asthma control is in studies that do not link to secondary care data^13,14^. While data on asthma-related hospitalisation from secondary care admission records is thought to be reproducible between studies^11,16^, records of asthma-related ED visits are felt to be much more poorly coded^9,11^. If ED data are to be useful, the quality and consistency of coding would need to improve.

While SABA overuse and exacerbation frequency can give an indication of asthma control, full evaluation requires assessment of self-report asthma symptom control and review of risk factors for poor asthma outcomes (Supplementary Table 4)^29^. Price et al. demonstrated that self-reported measures of asthma symptom control can be extracted from routinely collected patient-reported outcome measures^13,27,33^. The Royal College of Physicians’ 3 questions are a routinely collected assessment of asthma control in UK primary care as a criterion for QOF^34,35^. Given that this measure of symptom control should be available for all patients with asthma, this raises whether its inclusion would enhance the definition severe asthma when using primary care data.

Once a patient is identified as having asthma, is on high dose therapy and the asthma is uncontrolled, the next challenge is to differentiate severe asthma from DTA, where the poor asthma control is due to a modifiable factor (e.g. incorrect inhaler technique, poor adherence, smoking, comorbidities or an incorrect diagnosis). Primary care records can provide some of this information. Smoking status is another asthma QOF criterion, which should be available from the annual asthma review^26^. Primary care records can also provide information on comorbidities (Table 5). However, while certain conditions, including asthma, have benefitted from the increased quality of clinical coding from QOF, the quality of coding for other conditions outside QOF registers will vary significantly between practitioners. Prescription record data can give an indication on adherence through frequency of prescriptions. However, it gives no indication on whether the treatment is actually being taken or whether there is correct inhaler technique, and compliance with treatment may vary according to different psychosocial factors^8,20^.

There are a variety of potential opportunities if patients with severe asthma are more accurately identified. Patients with severe asthma have significantly better outcomes when identified in primary care and referred for specialist assessment^2^. For a subset of patients with severe asthma (severe eosinophilic asthma), new safe and effective management options are available, which can improve disease control and quality of life and reduce OCS burden. The remaining patients (severe non-eosinophilic asthma) have been shown to respond poorly to corticosteroids, and their ICS treatment can be reduced without an increase in exacerbation rates^36^. If we can identify these patients and their biomarker phenotype based on eosinophilia status, we could significantly reduce OCS burden and the associated side effect profile. In the UK, primary care data sets have near-to-complete population coverage of the background asthma population as the majority of citizens have a primary care record. The UK is uniquely placed to harness primary care records to identify patients with severe asthma at scale and reduce current inequalities in access and outcomes across geography, age, ethnicity and socio-economic groups^3,4^. The studies included in this review highlight how accurate identification of severe asthma could support research and planning, including better estimation of disease prevalence, clinical outcomes and healthcare resource utilisation.

To fully realise the potential of primary care data to identify and investigate patients with severe asthma, a number of challenges need to be overcome. International consensus is required on a standardised approach to defining asthma, asthma severity and asthma control when using these records. One of the major challenges identified throughout the study was the accuracy of primary care prescribing records. The use of OCS courses as a measure of acute exacerbations has limitations, and consideration should be given to whether incentivising better coding of acute asthma exacerbations in primary and secondary care records would give a more accurate measure of disease severity and control. Another key challenge for the identification of severe asthma identified throughout this review is the limited ability of prescription records from primary care to inform clinicians and researchers as to whether a patient is actually using prescribed treatment, and with the correct technique. Confirming adherence to maximal maintenance therapy is required to differentiate severe asthma from DTA. The extent to which SABA prescription records accurately represent poor control is largely determined by whether the numbers of prescribed inhalers accurately reflects a patient’s symptoms. This problem cannot be solved through linkage to other data sets. Novel approaches using smart inhalers are under investigation to assess treatment adherence and technique. The RASP-UK consortium demonstrated that data on adherence and technique from smart inhalers can inform decisions on when to step up treatment in severe asthma centres and identify patients whose inadequate symptom control may be a result of nonadherence rather than failure of inhaled treatment^36^.

This study focused on how routinely collected primary care data has been used to identify patients with severe asthma. This is extremely topical given the use of this data to identify patients with severe asthma as ‘high risk’ in the Coronavirus outbreak. Comparing the quantitative findings of the studies, including proportions of patients with varying levels of asthma severity and control was challenging as the data sets, cohort inclusion and exclusion criteria and definitions varied significantly between studies. Despite this variation, studies provided similar estimates, which can provide a baseline for further studies. Comparing the quality of reporting using the proportion of RECORD checklist points covered is not a validated approach, and results should be interpreted cautiously. However, we only included valid fields in estimates, and we believe it gives an overall indication of the quality of study reporting.

Primary care data are unique in its potential to represent the entire known asthma population. The coronavirus pandemic has placed a spotlight on the potential opportunities for clinical practice and research, which could be exploited if we can accurately identify severe asthma from primary care records. This review has highlighted a number of challenges that need to be overcome for an accurate diagnosis, including gaining consensus on a standardised approach to defining asthma, asthma severity and asthma control and ensuring the data accurately represent each component of the definition.

## Methods

The methodological approach was based on the Arksey and O’Malley framework for scoping reviews, which has been refined by Levac et al. and Pham et al.^37–39^. Scoping review methodology was chosen as our aim was to identify how research has been conducted and the knowledge gaps in this area^40^.

### Step 1: Identifying the research question

The research questions were (1) how has primary care data been used to identify and investigate severe asthma? (2) how does linkage to other healthcare and administrative data aids in this process? and (3) what was the quality of study reporting in articles using primary care data to identify patients with severe asthma?

### Step 2: Identification of relevant studies

Initial informal literature searches were carried out to identify terms used in the literature to investigate the use of primary care data to identify the prevalence and characteristics of severe asthma. A subject specialist medical librarian within Queen’s University Belfast advised on search terms required to ensure adequate coverage and retrieval of relevant studies (Supplementary Table 5). Formal literature searches were carried out in April 2020 on three databases: Embase, OVID Medline, and Web of Science. Minor adaptations in search terms were required to account for different database subject headings.

### Step 3: Study selection

The study selection process is summarised as a Preferred Reporting Items for Systematic Reviews and Meta-Analyses chart (Fig. 1). For a paper to be included, the following had to be true: (1) the primary care data or equivalent had to be collected as part of routine patient care and not collated for the specific purposes of a study; (2) data had to be from an entire primary care population, with the smallest unit being the known asthma population of a single primary care office; (3) the study had to identify varying levels of severity of asthma; and (4) the paper had to be a full peer reviewed article. Only studies published in English were included; abstracts, conference submissions and study protocol were excluded. Within the final list, we identified those articles that described linkage of records at an individual patient level. Studies that used linkage at aggregate level were excluded.

Article abstracts were screened (by J.S.) for eligibility using the above criteria. When insufficient information was available from the abstract to determine eligibility, articles were fully reviewed. When there was any doubt about inclusion, ambiguity was resolved after consensus discussion with another team member (F.K.).

### Step 4: Charting data

Data from the included articles were extracted and charted into a summary table. Data extracted from each article included characteristics of the data sets used, how asthma was defined, how asthma severity and control were defined and specific identified themes within the included articles, including characterisation of asthma cohorts, clinical outcomes, healthcare resource utilisation and quality of care. Key and recurring themes were identified in an iterative manner as each paper was reviewed. Following complete review of all articles, they were re-reviewed to ensure that all themes were captured.

### Step 5: Collating, summarising and reporting results

Data from the charting table were transferred into a summary table to enable comparison between articles.

### Evaluation of study reporting

We analysed the quality of reporting of each observational study against the RECORD Statement extension to the STROBE Statement checklist^21–23^.

### Reporting summary

Further information on experimental design is available in the Nature Research Reporting Summary linked to this paper.

## Supplementary information

Supplementary Information

Reporting Summary

## Data Availability

Any data generated or analysed are included in this article and the Supplementary Information files. Additional data may be available from the corresponding author on reasonable request.
