# Supplementary material for: Using routinely collected primary care records to identify and investigate severe asthma: a scoping review
Source: NPJ Prim Care Respir Med. 2021 Jan 26;31:1. doi: 10.1038/s41533-020-00213-9 (PMC7838272; doi:10.1038/s41533-020-00213-9)
Supplement: Supplementary file 1 — Supplementary Information [file 41533_2020_213_MOESM1_ESM.pdf]

Supplementary Table 1a – Estimates of asthma severity and control (Articles without linked data)

	GAYLE	PRICE 2015	TURNER	WALSH
SEVERITY ESTIMATES				
Treatment steps	BTS Steps<br>Children / Adults<br>4: 10.8% / 16.0%<br>5: 0.2% / 0.7%	BTS Steps<br>4: 22.4%<br>5: 0.9%	GINA Steps<br>4: 5.1%<br>5: 0.3%	BTS Steps<br>4: 3.6%<br>5: 1.0%
CONTROL ESTIMATES				
Overall measures of Control		Risk Domain[1]<br>Uncontrolled: 29.9%<br>Overall Control [2]<br>Uncontrolled: 59.3%<br>GINA [3 4]<br>Uncontrolled: 29.1%	Overall Control [5]<br>Uncontrolled = 26.7%	
Exacerbation frequency		See Table 4	See Table 4	OCS Courses<br>0: 87.5%<br>≥1: 12.5%
SABA use			Daily SABA Dosage (µg)<br>None: 21%<br>1-100: 12%<br>101-200: 28%<br>201-300: 17%<br>≥301: 22%	SABA Prescriptions<br>0-9: 86.4%<br>≥10: 13.6%

E: Eosinophil Count. BTS – British Thoracic Society. GINA – Global Initiative for Asthma. SABA – Short Acting Beta Agonist. ICS – Inhaled Corticosteroid.
[1] *Risk Domain Asthma Control - Absence of any acute respiratory event or asthma related outpatient visit in previous year*
[2] *Overall Asthma Control - Risk Domain Asthma Control (Absence of any acute respiratory event or asthma related outpatient visit in previous year) and ≤ 200µg salbutamol / 500µg terbutaline daily.*
[3] *GINA 2012 Control data were available for a subgroup with available GP record data (n = 13,552 (10.4%))*
[4] *GINA 2012 Uncontrolled Asthma - Three or more features of Partly Controlled Asthma which include daytime symptoms more than twice/week, any limitation of activities, any nocturnal symptoms/awakening, need for reliever treatment more than twice/week and <80% predicted lung function or personal best (Peak Flow or FEV_1).*
[5] *Controlled - No attack previous year and an average daily dose ≤ 200µg salbutamol / 500µg* terbutaline

Supplementary Table 1b - Estimates of asthma severity and control (Articles with linked data)

	YANG	NISSEN	BLOOM	PRICE2016	HULL	LARSSON	MOTH	SHIELDS
SEVERITY ESTIMATES								
Treatment steps	BTS Steps<br>COPD excluded (All)<br>4: 8.1% (11.4%)<br>5: 0.2% (0.2%)	BTS Steps<br>5: 12%<br>6: 0.9%	BTS Steps<br>Stratified by age provided in article	BTS Steps<br>4: 22.4%<br>5: 0.8%	BTS Steps<br>4: 2.3%<br>5: 0.2%	Swedish Guidelines [1]<br>(Based on GINA 2016)<br>Mild/Mod: 96.6%<br>Severe: 3.4%	Packages of anti-asthma medication<br>Mild (< 5): 48.8%<br>Mod (5-8): 36.0%<br>Severe (> 8): 15.2%	Utilisation suggestive of Moderate/Severe Asthma[3]: 3.08%
CONTROL ESTIMATES								
Overall measures of control						Poor control[2]<br>COPD excluded (All)<br>Mild/Mod: 26.5% / 44.2%<br>Severe: 50.8% / 58.6%		
Exacerbation frequency	See Table 3	See Table 3	See Table 3	See Table 3				
SABA use	SABA Prescriptions<br>COPD excluded (All)<br>0-12: 95.7% (94.8%)<br>≥13: 4.3 (5.2%)	SABA Prescriptions<br>0: 22.7%<br>1-2: 35.2%<br>3-9: 33.0%<br>≥10: 9.1%		Average SABA dosage (µg/day)<br>0 - 19.4%<br>1-200: 34.3%<br>201-400: 22.8%<br>≥400: 23.5%	SABA Prescriptions<br>0: 4.8%<br>1-3: 43.5%<br>4-12: 41.5%<br>≥13: 10.2%<br>Provided for 4 age groups in text	SABA ≥600<br>COPD excluded (All)<br>Mild/Mod: 14.0% / 26.3%<br>Severe: 27.7% / 30.1%		Beta-agonist overuse[4]<br>70.4%

BTS – British Thoracic Society. GINA – Global Initiative for Asthma. SABA – Short Acting Beta Agonist. ICS – Inhaled Corticosteroid.

[1] *Severe asthma: Collection of high dosage inhaled steroid (ICS, >800 budesonide or equivalent per day) and LTRA and/or LABA collected at the same time or within a 3-month period before or after the ICS. Patients who did not fulfil the severe asthma criteria were classified as having mild to moderate asthma.*

[2] *Poor Control: SABA ≥600 doses/year and/or ≥1 exacerbation during the year post index date.*

[3] *Utilisation suggestive of moderate / severe asthma: 3 asthma ED visits or ≥2 asthma hospitalisations.*

[4] *Beta-agonist overuse: >6-month supply in 6-month period.*

Supplementary Table 2a – Estimates of Clinical Outcomes (Articles without linked data)

	GAYLE	PRICE 2015	TURNER	WALSH
OUTCOME ESTIMATES				
Exacerbation rates				
Exacerbations (using combined measure)		ATS / ERS Definition<br>Total<br>0: 80.8%<br>1: 12.3%<br>2-3: 4.9%<br>≥4: 2.0%	ATS / ERS Definition<br>0: 83.1%<br>1 = 12.2%<br>≥2 = 4.7%	
OCS prescriptions		OCS Courses<br>Total<br>0: 84.0%<br>1: 11.9%<br>≥2: 4.1%		OCS Courses<br>0: 87.5%<br>≥1: 12.5%
ED visit				
Hospitalisation				
GP visit				
Asthma death				

ATS/ERS - American Thoracic Society / European Respiratory Society. ED – Emergency Department. GP – General Practice. OCS – Oral Corticosteroid. SABA – Short Acting Beta Agonist.

Supplementary Table 2b – Estimates of Clinical Outcomes (Articles with linked data)

	**YANG**	**NISSEN**	**BLOOM**	**PRICE2016**	**HULL**	**LARSSON**	**MOTH**	**SHIELDS**
OUTCOME ESTIMATES								
Exacerbation rates		Exacerbation Rate / 1000 person years<br>Provided for 6 phenotypes in text	Exacerbation Rate / 10 person years<br>Age ≤ 5: 4.27<br>Age 5-17: 1.48<br>Age 18-54: 3.22<br>Age ≥55: 9.40					
Exacerbations (using combined measure)			Proportion with ≥1 exacerbation / year<br>Age ≤ 5: 21.4%<br>Age 5-17: 22.3%<br>Age 18-54: 38.0%<br>Age ≥55: 62.5%	ATS / ERS Definition<br>0-1: 93.1%<br>≥2: 6.9%		ATS / ERS Definition<br>≥1 exacerbation<br>Mild/Mod: 16.2%<br>Severe: 36.3%		
OCS prescriptions	≥2 OCS courses<br>COPD excluded: 5.1%<br>All: 7.0%			OCS courses<br>1: 10.9%<br>2: 3.8%<br>≥3: 4.2%		≥1 OCS courses<br>Mild/Mod: 16.0%<br>Severe: 36.3%		Systemic Corticosteroids<br>Mean / year: 0.56
ED visits	≥1 ED attendance or hospital admission<br>1.2%		ED visits<br>Provided for 4 age categories in text	ED Visits:<br>≥1: 0.3%		≥1 ED Visit<br>Mild/Mod: 0.1%<br>Severe: 0.9%		ED visits<br>Mean / year: 0.0.2
Hospitalisations			Hospitalisations<br>Provided for 4 age categories in text	Hospitalisations<br>≥1: 0.3%	Hospitalisation Rate<br>/ 100 population<br>Total: 2.43<br>Provided for 4 age categories in text	≥1 Hospitalisation<br>Mild/Mod: 0.3%<br>Severe: 1.2%		Hospitalisations<br>Mean / year: 0.06
GP visits					GP Visits<br>Median number: 3<br>Provided for 4 age categories in text	≥1 GP Visits<br>Mild/Mod: 21.8%<br>Severe: 27.4%		Asthma Visits<br>Mean / year: 1.37
Asthma deaths			Asthma Deaths<br>Provided for 4 age categories in text					

ATS/ERS - American Thoracic Society / European Respiratory Society. ED – Emergency Department. GP – General Practice. OCS – Oral Corticosteroid SABA – Short Acting Beta Agonist.

Supplementary Table 3 - Asthma Severity Definitions

	BTS 2011	BTS 2014	BTS 2016	BTS 2019	GINA 2012	GINA 2017	GINA 2019	Coronavirus Pandemic Severe Asthma Definition
Step 1	Inhaled SABA as required	Inhaled SABA as required	Low dose ICS	Low dose ICS	As needed Rapid acting β2 agonist	Mild Asthma<br>Consider low dose ICS	As needed low dose ICS-fomoterol<br><br>Low dose ICS (is SABA as reliever)	
Step 2	Add ICS 200-800 mcg/day	Add ICS 200-800 mcg/day	Add inhaled LABA to low-dose ICS (normally as a combination inhaler)	Add inhaled LABA to low-dose ICS (normally as a combination inhaler)	Low dose ICS<br>or<br>LTRA	Mild Asthma<br>Low dose ICS<br>or<br>LTRA	Daily low dose ICS<br>or<br>As needed low dose ICS-fomoterol<br><br>LTRA or Low dose ICS (if SABA as reliever)	
Step 3	Add inhaled LABA<br><br>Assess control of asthma:<br>• good response to LABA - continue LABA<br>• benefit from LABA but control still inadequate continue LABA and increase ICS dose to 800 mcg/day<br>• no response to LABA - stop LABA and increase ICS dose to 800 mcg/ day<br><br>If control still inadequate, institute trial of LTRA or SR theophylline	Add inhaled LABA<br><br>Assess control of asthma:<br>• good response to LABA - continue LABA<br>• benefit from LABA but control still inadequate continue LABA and increase ICS dose to 800 mcg/day<br>• no response to LABA - stop LABA and increase ICS dose to 800 mcg/ day<br><br>If control still inadequate, institute trial of LTRA or SR theophylline	• No response to LABA – stop LABA and consider increased dose of ICS<br>• Benefit from LABA but control still inadequate - continue LABA and increase ICS to medium dose<br>• Benefit from LABA but control still inadequate – continue LABA and ICS and consider trial of other therapy – LTRA, SR theophylline, LAMA	Consider:<br>• Increasing ICS up to medium dose<br>• Adding LTRA<br><br>If no response to LABA, consider stopping LABA	Low dose ICS plus LABA<br>or<br>Medium or high dose ICS plus<br>or<br>Low dose ICS plus LTRA<br>or<br>Low dose ICS plus theophylline SR	Moderate Asthma<br>Low Dose ICS/LABA	Low dose ICS plus LABA<br>or<br>Medium dose ICS<br>or<br>Low dose ICS plus LTRA	
Step 4	Consider trials of:<br>• Increasing ICS up to 2000 mcg/day<br>• Add fourth drug e.g. LTRA, SR theophylline, β2 agonist tablet	Consider trials of:<br>• Increasing ICS up to 2000 mcg/day<br>• Add fourth drug e.g. LTRA, SR theophylline, β2 agonist tablet	Consider trials of:<br>• Increasing ICS up to high dose<br>• Addition of a fourth drug, eg LTRA, SR theophylline, beta agonist tablet, LAMA<br><br>Refer for specialist care	Refer patient for specialist care	Medium or high dose ICS plus LABA<br><br>And/or a third controller - LTRA or Theophylline SR	Severe Asthma<br>Medium/High Dose ICS/LABA<br>or<br>High dose ICS plus second controller	Medium dose ICS plus LABA<br>Or<br>High dose ICS plus add on LAMA or LTRA	All 3 of:<br>•ICS (at any dose)<br>•another preventer LABA or LTRA<br>•regular or continuous OCS (4 or more prescriptions July and December 2019)<br>or<br>Hospital admission in the last 12 months for your asthma<br>or<br>Ever admitted to an intensive care unit for asthma
Step 5	Daily OCS – Lowest dose possible<br><br>Maintain high dose ICS at 2000 mcg/day<br><br>Refer patient for specialist care	Daily OCS – Lowest dose possible<br><br>Maintain high dose ICS at 2000 mcg/day<br><br>Refer patient for specialist care	Use daily OCS - lowest dose providing adequate control<br><br>Maintain high dose ICS<br><br>Refer patient for specialist care		OCS<br><br>Anti IgE Treatment	Severe Asthma<br>High dose ICS/LABA plus oral corticosteroids<br>or<br>High dose ICS/LABA plus other agents	High dose ICS plus LABA<br><br>Refer for specialist assessment and add on therapy (LABA or monoclonal antibody)<br><br>Low dose OCS	

BTS – British Thoracic Society. GINA - Global Initiative for Asthma. SABA - Short Acting Beta Agonist. ICS – Inhaled Corticosteroid. LABA Long Acting Beta Agonist. LTRA – Leukotriene Receptor Antagonist. SR – Sustained Release. OCS – Oral Corticosteroid. LAMA – Long Acting Muscarinic Antagonist.

Supplementary Table 4 – Asthma Control Definitions

	GINA 2012	GINA 2017	RCP 3 Questions
Uncontrolled	A - Asthma current clinical control (Preferable over 4 weeks)<br>Uncontrolled defined by three of more features of:<br>• Daytime symptoms more than twice/week<br>• Any limitation of activities<br>• Any nocturnal symptoms<br>• Need for reliever treatment more than twice per week<br>• Lung function (PEF or FEV1) <80% predicted<br><br>B – Assessment of Future Risk<br>Features associated with increased risk of adverse events<br>• Poor clinical control<br>• Frequent exacerbations in past year<br>• Ever admission to critical care for asthma<br>• Low FEV1<br>• Exposure to cigarette smoke<br>• High dose mediations	A - Asthma Symptom Control<br>Uncontrolled defined by 3-4 features in the past 4 weeks of:<br>• Daytime symptoms > twice/week?<br>• Night waking due to asthma?<br>• Reliever needed for symptoms > twice/week?<br>• Activity limitation due to asthma?<br><br>B - Risk factors for poor asthma outcomes<br>Potentially modifiable risk factors<br>• Uncontrolled asthma symptoms<br>• High SABA use<br>• Inadequate ICS<br>• Low FEV1<br>• Psychological/socioeconomic problems<br>• Exposures (Smoking or allergens)<br>• comorbidities<br>• sputum or blood eosinophilia<br>• pregnancy<br>Major Independent risk factors<br>• Every intubates or ICU for asthma<br>• ≥severe exacerbation last 12 months	1. Have you had difficulty sleeping because of your asthma symptoms (including cough)?<br><br>2. Have you had your usual asthma symptoms during the day (cough, wheeze, chest tightness or breathlessness)?<br><br>3. Has your asthma interfered with your usual activities (for example housework, work or school)?"

GINA – Global Initiative for Asthma. RCP – Royal College of Physicians. SABA – Short Acting Beta Agonist. PEF – Peak Expiratory Flow. FEV_1 – Forced Expiratory Volume in the first second. ICU – Intensive Care Unit.

Supplementary Table 5 – Search Terms

1	asthma/ or asthma, aspirin-induced/ or asthma, exercise-induced/ or asthma, occupational/ or status asthmaticus/
2	severe* asthma*.mp.
3	severe refractory asthma*.mp.
4	difficult asthma*.mp.
5	Medical Records Systems, Computerized/ or Electronic Health Records/ or Medical Records/
6	record*.mp.
7	data*.mp.
8	registries/
9	regis*.mp.
10	1 or 2 or 3 or 4
11	5 or 6 or 7 or 8 or 9
12	10 and 11
13	primary health care/
14	general practice/ or family practice/
15	Primary Care.mp
16	13 or 14 or 15
17	12 and 16
